# Age-Related Differences in Susceptibility to Carcinogenesis. II. Approaches for Application and Uncertainty Analyses for Individual Genetically Acting Carcinogens

**DOI:** 10.1289/ehp.7564

**Published:** 2005-01-10

**Authors:** Dale Hattis, Robert Goble, Margaret Chu

**Affiliations:** ^1^George Perkins Marsh Institute, Clark University, Worcester, Massachusetts, USA; ^2^Office of Research and Development, U.S. Environmental Protection Agency, Washington, DC, USA

**Keywords:** carcinogenesis, fetal, mutagenic chemicals, risk assessment, susceptibility, uncertainties

## Abstract

In an earlier report we developed a quantitative likelihood-based analysis of the differences in sensitivity of rodents to mutagenic carcinogens across three life stages (fetal, birth to weaning, and weaning to 60 days) relative to exposures in adult life. Here we draw implications for assessing human risks for full lifetime exposures, taking into account three types of uncertainties in making projections from the rodent data: uncertainty in the central estimates of the life-stage–specific sensitivity factors estimated earlier, uncertainty from chemical-to-chemical differences in life-stage–specific sensitivities for carcinogenesis, and uncertainty in the mapping of rodent life stages to human ages/exposure periods. Among the uncertainties analyzed, the mapping of rodent life stages to human ages/exposure periods is most important quantitatively (a range of several-fold in estimates of the duration of the human equivalent of the highest sensitivity “birth to weaning” period in rodents). The combined effects of these uncertainties are estimated with Monte Carlo analyses. Overall, the estimated population arithmetic mean risk from lifetime exposures at a constant milligrams per kilogram body weight level to a generic mutagenic carcinogen is about 2.8-fold larger than expected from adult-only exposure with 5–95% confidence limits of 1.5-to 6-fold. The mean estimates for the 0- to 2-year and 2- to 15-year periods are about 35–55% larger than the 10- and 3-fold sensitivity factor adjustments recently proposed by the U.S. Environmental Protection Agency. The present results are based on data for only nine chemicals, including five mutagens. Risk inferences will be altered as data become available for other chemicals.

Both the Safe Drinking Water Act (SDWA) Amendments (1996) and the Food Quality Protection Act (FQPA 1996) direct U.S. Environmental Protection Agency (EPA) to conduct studies to identify and characterize health risks for groups that may be at greater risk than the general population. For non-cancer health effects, the FQPA (but not the SDWA) mandates the use of a default additional 10-fold safety factor (10×) for protection of children from noncancer effects unless specific data are available to indicate that this extra protection is not needed. For carcinogenic risks, as part of its revision of cancer risk assessment guidelines, the U.S. EPA has assembled and analyzed animal cancer bioassay data for exposures to mutagenic and putatively nonmutagenic chemicals over different periods of life. On the basis of this analysis, the U.S. EPA proposed a 10-fold upward adjustment in the cancer potency for exposures to mutagenic carcinogens from birth to 2 years of age and a 3-fold adjustment for exposures between 3 and 15 years of age (U.S. EPA 2003).

In a previous report (Hattis et al. 2004), we offered an improved analysis of the available cancer bioassay data, using likelihood methods to avoid excluding cases where no tumors were observed in either adult or other groups and providing for quantitative estimation of confidence limits for the data as a whole, and selected subsets of the data. We expressed dosage for animals of different weights on a metabolically consistent basis (either concentration in air or food, or per unit body weight to the three-quarters power). Finally, we used a system of dummy variables to represent exposures during fetal, preweaning, and weaning to 60-day postnatal periods—yielding separate estimates of relative sensitivity per day of dosing in these intervals.

Briefly, the central estimate results of that analysis indicated a 5- to 60-fold increased carcinogenic sensitivity in the birth to weaning period per dose/(body weight^3/4^-day) for mutagenic carcinogens, and a somewhat smaller increase—centered about 5-fold—for radiation carcinogenesis per Gray (100 rads). Effects were greater in males than in females, partly because of considerable differences in the carcinogenic responsiveness of the liver in males. There was a similar increased sensitivity in the fetal period for direct-acting nitrosoureas, but no such increased fetal sensitivity was detected for carcinogens requiring metabolic activation.

This present article is a follow-up to that earlier work (Hattis et al. 2004) showing how the previous results might be applied to distributional risk analyses of specific mutagenic carcinogens. Doing this requires analyses of three particular sources of uncertainty: *a*) uncertainty in the central estimates of the life-stage–specific sensitivity factors estimated earlier, *b*) uncertainty from chemical-to-chemical differences in life-stage–specific sensitivities for carcinogenesis, and *c*) uncertainty in the mapping of rodent life stages to human ages/exposure periods. The implications of these three component uncertainties are assessed in Monte Carlo simulations.

Methodology and results from assessing each source of uncertainty separately are covered in the next three subsections. This is followed by a discussion of methodology and results from the Monte Carlo simulations of the combined effects. To convey our methods and results as transparently as possible, and allow others to extend the analyses, the underlying distributional input data and the Excel Monte Carlo simulation models for each sex are available via our website (Hattis 2004). The results provide guidance on *a*) implications for human risk assessment for full lifetime exposures relative to adult-only exposures, with comparisons with the human relative susceptibility assumptions in the U.S. EPA (2003) proposal and *b*) implications for research priorities to reduce uncertainties.

## Uncertainties in Central Estimates of Life-Stage–Specific Sensitivity to Carcinogenesis for Mutagenic Agents

**Figure 1:**
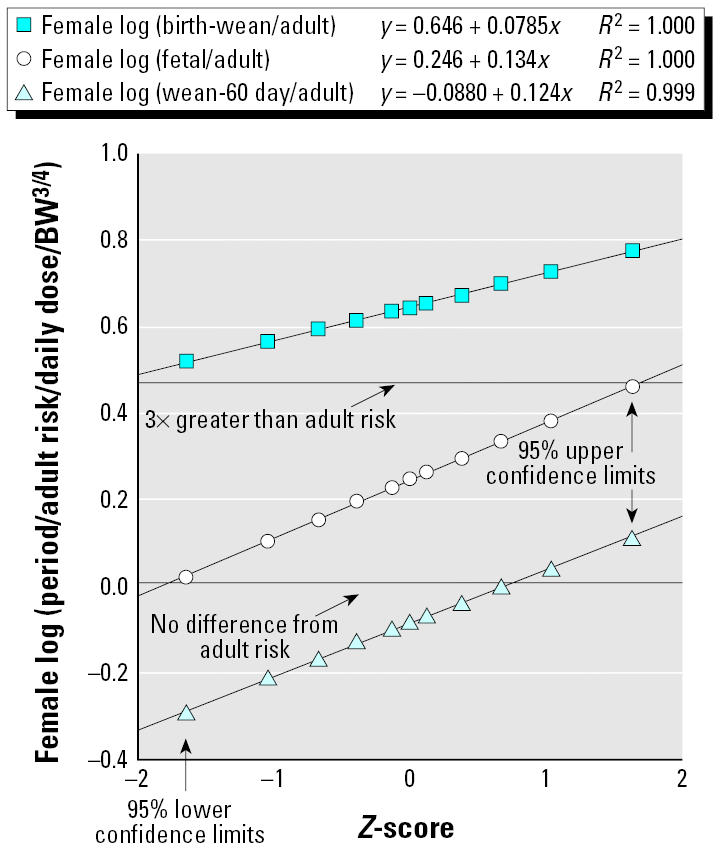
Females: log-normal plots of likelihood-based uncertainty distributions in rates of cancer transformations per daily dose for various life stages for mutagenic chemicals (relative to comparable exposures of adults) for combined discrete and continuous dosing experiments. BW, body weight.

**Figure 2:**
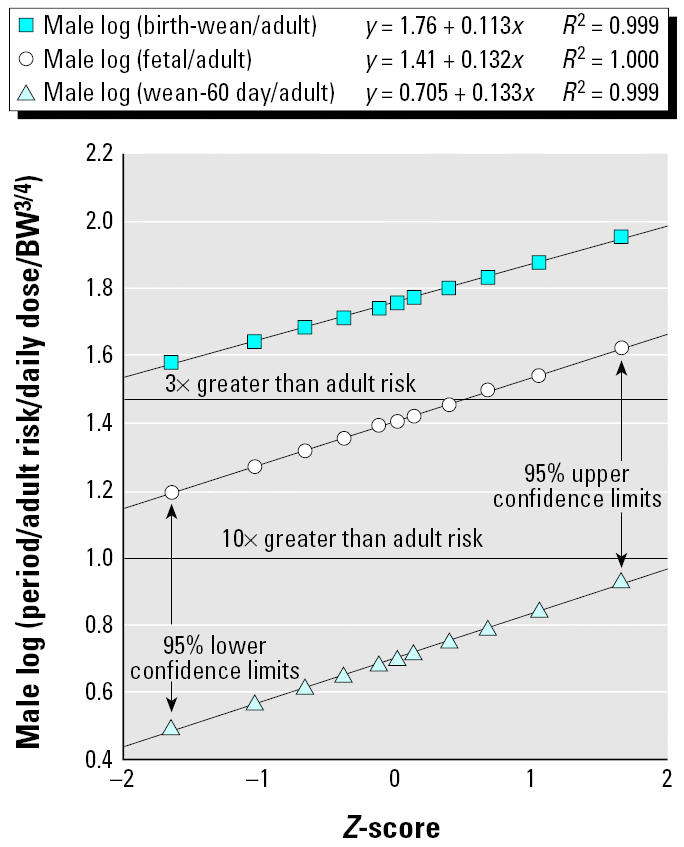
Males: log-normal plots of likelihood-based uncertainty distributions in rates of cancer transformations per daily dose for various life stages for mutagenic chemicals (relative to comparable exposures of adults) for combined discrete and continuous dosing experiments. BW, body weight.

Table 1 and Figures 1 and 2 show the results of our prior analyses (Hattis et al. 2004) of the overall central tendency differences between exposures during various life stages and similar exposure (per unit body weight^3/4^ or per unit concentration in external air or water or food) per day during adulthood (> 60 days of age in rodents). There are appreciable differences between the estimated life-stage–specific increments in relative risk for the two sexes; therefore, all the analyses in this article are done separately for males and females.

The origins of the sex difference are not known; however, we note that there are conspicuous differences between male and female rodents in the levels of cytochrome P450 (CYP) enzymes that are responsible for metabolic activation of several small-molecular-weight mutagenic carcinogens. For example, Chanas et al. (2003) have recently observed a greater than 5-fold difference in CYP2E1 levels in male than in female adult mice, and associated this with an enhanced male sensitivity to the toxicity of acrylonitrile. Early-life differences in expression of specific CYPs have been associated with sex differences in the frequency of growth hormone pulses observed in the plasma of rodents (Pampori and Shapiro 1994; Pampori et al. 2001; Shapiro et al. 1995). Sex differences are also apparent in the induction of some DNA repair enzymes *in vivo* in rodent liver. For example, Chan et al. (1992) observed over a 17-fold induction of O^6^-methyl-guanine methyl transferase activity in the livers of female Sprague-Dawley rats after a high-dose (15 Gy) gamma radiation exposure; this is compared with a much smaller 3.5-fold induction in the livers of male rats. Similar sex-related differences were not observed in other organs. We are not aware of direct comparisons of the induction of such DNA repair functions between infant/juvenile and adult animals.

Figures 1 and 2 show log-normal probability plots (Hattis and Burmaster 1994) of the statistical uncertainty distributions for the life stage/adult sensitivity ratios for the male and female combined discrete and continuous dosing data for mutagenic carcinogens. In this type of plot, correspondence of the points to the fitted line is an indicator of the fit of a log-normal distribution to the statistical uncertainties in central estimate life stage/adult sensitivity ratios. (The *Z*-score that makes up the *x*-axis is the number of SEs above or below the median of the normal distribution of log_10_-transformed values). Figures 1 and 2 show that the uncertainty distributions from the overall fits to the data are well described by fitted log-normal distributions. We stress that these plots are of confidence limits on the aggregate geometric mean results for all chemicals in the covered groups.

## Departures from Life-Stage–Specific Model Fits from the Central Estimates for Individual Chemicals

A risk assessor or risk manager considering the risks of exposure to a particular carcinogen faces more uncertainty than the simple statistical confidence limits on the aggregate fit of all the data quantified in the preceding section. There is also the chance that the particular chemical under study differs in its relative life-stage–specific/adult sensitivity ratios from the geometric mean of other chemicals in the group providing observational data.

**Figure 3:**
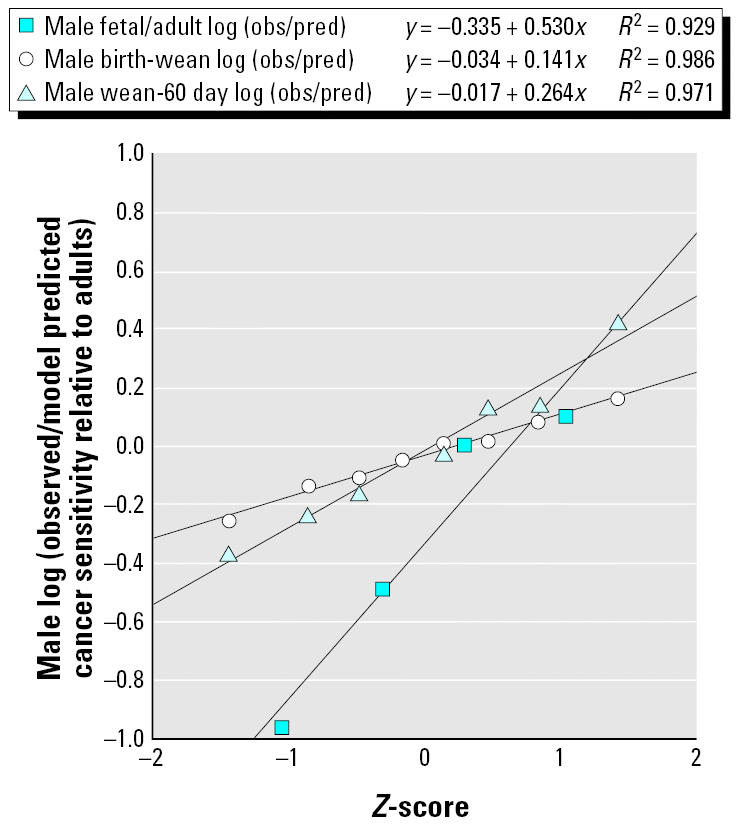
Males: probability plots of the individual chemical geometric mean ratios of observed/geometric mean model predicted excess cancer transformations over control tumor rate/(dose/kg body weight^3/4^) for treatment in various life stages relative to adults.

**Figure 4:**
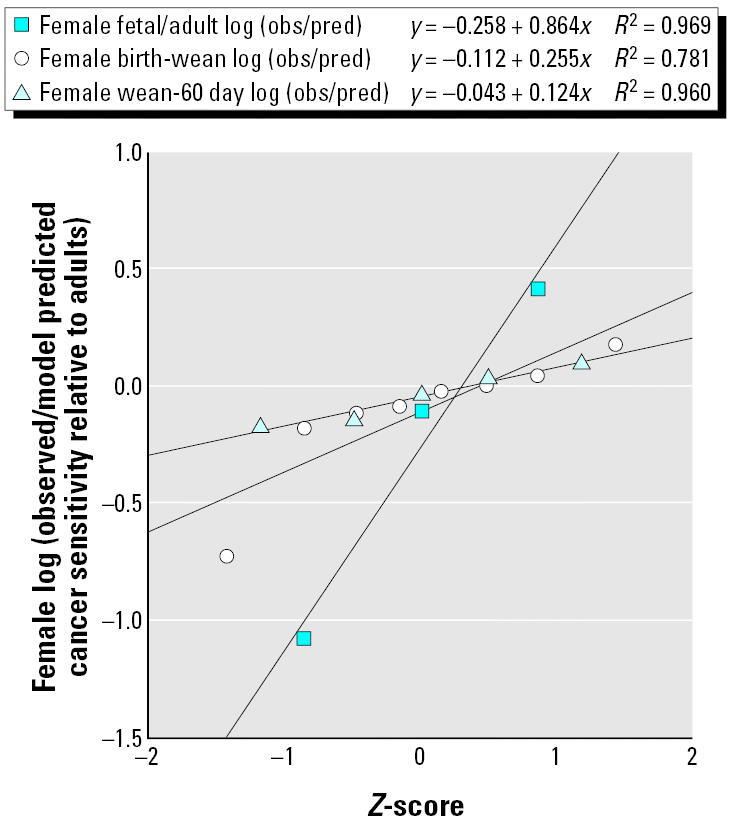
Females: probability plots of the individual chemical geometric mean ratios of observed/geometric mean model predicted excess cancer transformations over control tumor rate/(dose/kg body weight^3/4^) for treatment in various life stages relative to adults.

To give assessors and managers a preliminary set of estimates of chemical-to-chemical differences, Table 2 shows an analysis of the subset of the life-stage–specific carcinogenesis data where groups of animals received exposure that was confined to a single life stage (i.e., fetal, birth to weaning, or weaning to 60-day periods). Other data points contributing to the fits in Table 1 had exposures that extended across various life stages, were for adults only, or were unexposed controls. At the bottom of the table are standard deviations of the common logarithms (using a base of 10) of the departures of the chemical-specific observations from the overall model predictions. In the later Monte Carlo simulations, the antilog of this factor will be used as the geometric standard deviation of a log-normally distributed multiplier for the life-stage–specific risks with a geometric mean of 1. Figures 3 and 4 show that, although the data are sparse, log-normal distributions are generally reasonable descriptions of these data.

The limited data for the fetal life stage also suggest greater chemical-to-chemical differences than are present for the birth to weaning and weaning to 60-day exposure periods (Table 2). Observations in the previous report indicated that there were substantial differences between direct-acting chemicals (nitrosoureas) and chemicals requiring metabolic activation in the extent of elevation of fetal-stage carcinogenesis sensitivity over the sensitivity to exposures during adulthood.

The approach represented here is not the only possible way in which chemical-to-chemical differences might have been analyzed. In some ways a better approach might have been to estimate all of the coefficients and uncertainties shown in Table 1 separately for each chemical and sex. Had that been possible, we could have preserved for the Monte Carlo simulations whatever dependencies there might have been in the data between life-stage–specific risk increments for individual chemicals. Unfortunately, this would have required estimates of five different parameters per sex per chemical per tumor site (the background rate of tumors, the tumor risk for adult-only exposure, and the relative multiplicative increment of tumor risk for each of the three life stages). After attempting this for a few chemicals, we concluded that few if any of the chemicals and tumor sites for which we had information had rich enough data sets to support robust estimation of the required five independent parameters.

As an alternative, to check for dependencies we did simple pairwise correlation analyses of the data in Table 2 for different life stages. Of the six possible pairwise correlations, we found only one that was marginally statistically significant at *p* < 0.05—a finding that could easily be the result of chance fluctuations and multiple comparisons. We therefore elected not to incorporate this possible dependency into our Monte Carlo simulation analysis of uncertainties in overall life stage–specific risks.

## Mapping Rodent Life Stages to Human Periods: Implications for Uncertainties in Projections of Expected Risks for Lifetime Exposures to Mutagenic Carcinogens

A U.S. EPA committee (Brennan et al. 2003) previously defined a series of human age groups based on behavioral and physiologic milestones likely to predict changes in exposure rates (Table 3). Unfortunately, it is not clear how these proposed divisions relate to the fetal, birth to weaning, and weaning to 60-day periods used in our previous analysis of excess risks from rodent early-life exposures to mutagenic carcinogens. Ideally, a theory for interspecies mapping of differences in the timing of enhanced susceptibility for carcinogenesis should be based on an understanding of the carcinogenic process, and how it is affected by age.

**Figure 5:**
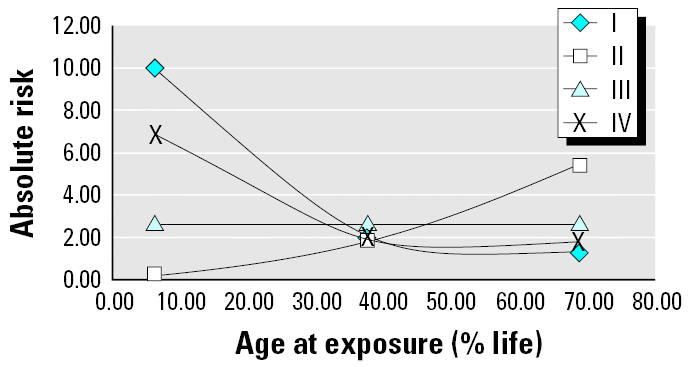
Effects of age at exposure on risks over background for classical Armitage-Doll five-stage multistage models in which the carcinogen enhances different transitions over a background lifetime risk of 10%: I, enhancement of the first stage only; II, enhancement of the fourth stage only; III, equal enhancement of all stages (Pierce and Mendelsohn 1999); IV, smoking-radon analogy two-thirds enhancement of stage I and one-third enhancement of stage 4.

Considerable past work has emphasized the potential for age-related differences in long-term risks from carcinogenic exposures that could result from early- versus late-life exposure to carcinogens that tend to cause mutations at a single stage that is either early or late in the multistage molecular pathologic sequence of genetic changes (Brown and Hoel 1986; Day and Brown 1980; Whittemore 1977). In general, carcinogenic risks will tend to be greater for early-life exposure to a carcinogen that causes relevant early-stage transitions but will tend to be greater for late-life exposure to a carcinogen that causes relevant late-stage transitions. For example, Figure 5 shows the effects of age at exposure on absolute excess risks against a 10% lifetime background cancer for classical five-stage Armitage-Doll models in which different stage transitions are enhanced by a carcinogenic exposure.

Recent analyses of atomic bomb survivor data have tended to de-emphasize this type of mechanistic consideration. Analyses by Pierce and Mendelsohn (1999) suggest that those data are most compatible with a model in which radiation enhances all stages of classical Armitage-Doll processes (model III in Figure 5). If this is correct, although excess relative risks are much greater for early-life exposures in the first decades after exposure, eventual lifetime absolute risks per dose (the types of estimates made by the U.S. EPA (1999) in its typical slope factor assessments) are expected to be much less influenced by age at exposure. The most recent empirical excess absolute risk descriptions from the atomic bomb survivor data (Preston et al. 2003) appear to project lifetime absolute risks that are only about twice as large for exposure before 15 years of age than for exposure between 15 and 60 years of age. It is not clear, however, that these data have been analyzed for very fine breakdowns of early-life human exposures (i.e., finer than 10-year age periods, such as 0–9 years); 90% of the people in the atomic bomb survivor group who were exposed as 0- to 9-year-olds are still alive, so it is likely that much more extensive examination of the eventual cancer mortality experience of the youngest exposed people will be possible in the next few decades.

One plausible factor that may be contributing to life-stage–specific differences in risks of carcinogenic transformation per unit dose is a difference in cell replication rates for relevant stem cells. During early life stages, it is likely that these cells reproduce more quickly to support the generation of additional cells at all stages of differentiation that are required to make up the growing organism. Because of more rapid reproduction of such stem cells, there is likely to be less time to accomplish DNA repair before copying and the fixation of newly generated DNA lesions into permanent point mutations and larger chromosomal changes. Therefore, it is natural to attempt to make some estimates of equivalent times in different species that are related to some measures of growth in those species.

**Figure 6:**
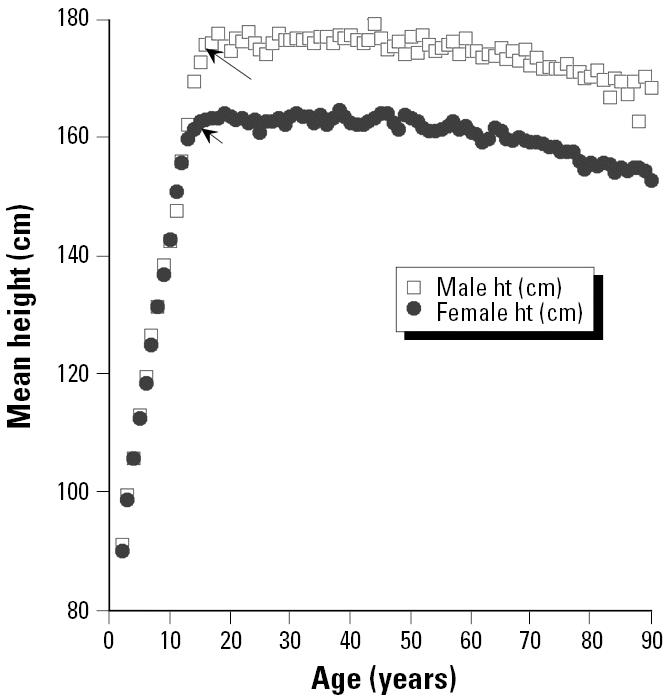
Population-weighted differences in mean height (ht) for NHANES III subjects of different ages (2–90 years).

**Figure 7:**
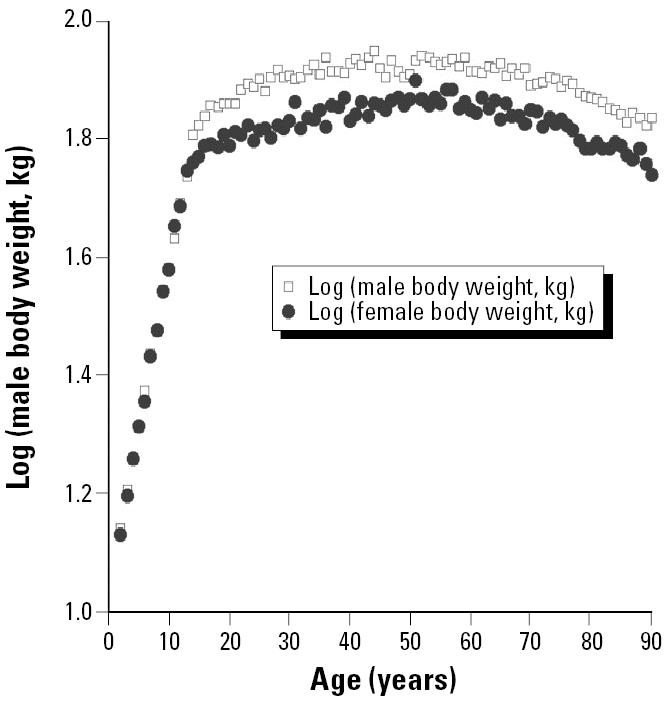
Population-weighted differences in log(mean weight in kg) for NHANES III subjects of different ages (2–90 years).

**Figure 8:**
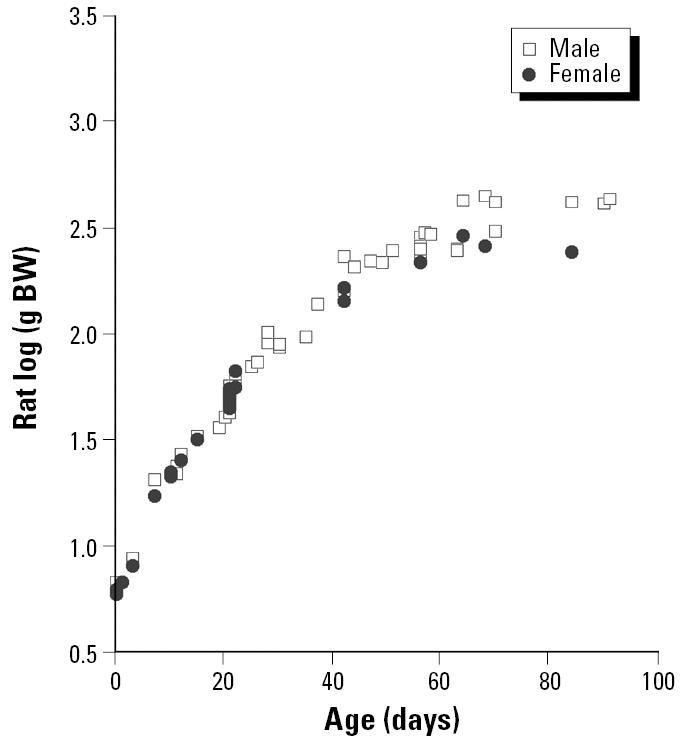
Postnatal growth of Sprague-Dawley rats, based on data compiled for the U.S. EPA. Data from Gentry et al. (2003). BW, body weight.

**Figure 9:**
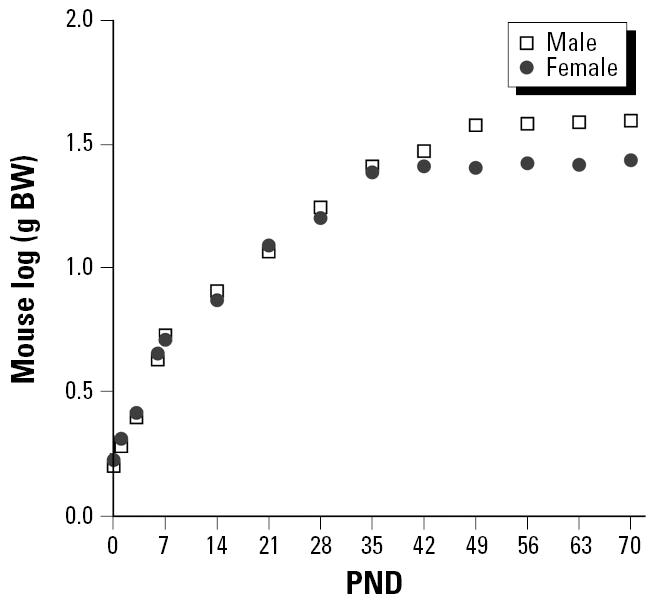
Postnatal growth of ICR/Jcl mice, based on data from Nomura (1976). Abbreviations: BW, body weight; PND, postnatal day.

All measures of growth, of course, are not equally likely to be accurate reflections of the kind of stem cell replication that is likely to lead to increases in vulnerability to carcinogenesis. Figures 6 and 7 contrast two measures of growth—body weight versus height—that are available for a large representative sample of U.S. humans [from the Third National Health and Nutrition Examination Survey (NHANES III); National Center for Health Statistics 1996]. Although growth in height for average people ceases fairly abruptly at 15 or 16 years of age (depending on sex), average weights of U.S. humans continue to increase well into middle age. Rats apparently show a similar pattern of continual increase in weight well into adulthood in standard National Toxicology Program bioassay studies (NTP 1999). Unfortunately, we were not able to locate measurements of linear growth in rodents that might provide more sharply defined points of comparison for the data in Figure 6. We were, however, able to obtain data sets for body weight covering the post-natal (and in some cases prenatal) developmental periods (Figures 8 and 9).

Failing comparable measurements of linear growth, we elected to anchor our weight-related estimates of relative age to another type of developmental milestone that occurs near the age where “adulthood” is generally defined—sexual maturity. Table 4 gives data from a recent report (Kilborn et al. 2002) describing times of the onset of sexual maturity in different species. Using these developmental anchor points, Tables 5 and 6 show the fraction of sexual maturity body weights achieved at the borders between the various rodent exposure periods used in the prior analysis, and the ages at which average humans of each sex achieve the same fractions of sexual-maturity body weights. The human ages corresponding to rodent weaning (assumed to be 21 postnatal days in both mice and rats) show a large variation between projections from mouse versus rat data, and within each rodent species between males and females. This necessarily leads to substantial uncertainties in alternative estimates of the amount of human time that would correspond to the birth to weaning period in particular (Table 7). The length of this interval is critical for the analysis here because the birth to weaning period shows the greatest increase in relative risk per dose per day of exposure (Table 1), and the implications for lifetime relative risk depend directly on how large a part of the life span is covered by the “birth to weaning” risk elevation per unit daily dose/body weight^3/4^.

It is a substantial challenge to fairly represent this uncertainty in a Monte Carlo analysis. Some other analysts, faced with two estimates of an uncertain quantity, have chosen to represent the uncertainty with uniform distributions with limits defined by the two points. Our view is that this generally understates the associated uncertainty because there can be no assurance that the two available estimates happen to represent the absolute lowest and highest possible values for the uncertain parameter. We believe that sharp limits on uncertainty distributions should be set only where there is good reason to believe that values outside the limits are impossible (Hattis and Burmaster 1994). For the present case, we find it hard to believe that the human equivalent of the total period from birth through 60 days in rodents could be more than about 15 years in females or 16 years in males—corresponding to the average cessation of vertical growth seen in the NHANES III data (Figure 6). We therefore chose to define log-normal uncertainty distributions as shown in Table 7 for human equivalents of the various rodent exposure periods, subject only to the limitation that within any Monte Carlo trial, the total birth to weaning plus weaning to 60-day equivalents could not exceed these sex-dependent limits. In cases where these limits were exceeded on individual trials, both the component periods were reduced proportionately to values that would add up to the prescribed limits. This introduced a negative dependency between possible values for the birth to weaning and weaning to 60-day periods.

## Monte Carlo Simulation Modeling of the Uncertainties in Full-Life Exposures to a Generic Mutagenic Carcinogen

Using the Microsoft Excel rand( ) and norm-sinv( ) commands, (Microsoft Excel X for Mac, Microsoft Corp., Redmond, WA) each simulation trial drew random values for a particular sex for the central estimate of the risk/dose multiplier for each of the three periods relative to adults (see Figures 1 and 2 for log-normal parameters), the chemical-to-chemical relative risk multiplier [geometric mean of 1 and log(geometric standard deviations) in Table 2], and the length of the human equivalents of the three periods, subject to the 15- and 16-year limitations described above.

The period-specific increments to lifetime risk (relative to comparable adult period exposure, defined as 1) were then calculated as the product of these three terms normalized to the calculated duration of the adult period for that trial. (The length of the adult period varied from trial to trial as the difference between 70 years and the sum of the human-equivalent birth to weaning and weaning to 60-day periods.) The model spreadsheets available on the website (Hattis 2004) should be consulted for further methodologic details.

The uncertainty distributions for the sex-specific and life-stage–specific contributions to expected lifetime risk are given in Table 8. In each case the numbers represent the increment to lifetime relative risk/dose where the risk from treatment for the full adult period is defined as 1. For example, the 50th percentile of the uncertainty distribution under the “male fetal” column is 0.173. This means that treatment at the similar dose rate to the mother through the fetal period (rodent gestation day 12 equivalent through birth) is expected to produce about 17% of the lifetime risk of exposure to the generic mutagenic carcinogen through the entire period of adulthood.

The potential aggregate public health significance of these results can be seen in the “bottom line” distributions provided in Table 9. The final column, aggregating results for males and females, suggests that full lifetime risks for full life constant exposure per kilogram of body weight^3/4^ to a generic mutagenic carcinogen are expected to be about 3.5 times larger than would be estimated for similar exposure only through the full period of adulthood. There is appreciable uncertainty in this estimate (with 5–95% confidence limits corresponding to a range from a 60% increment to nearly an 8-fold increment from adult-only exposure), but it gives analysts and decision makers a starting point for reasoning about the potential risks from early-life exposures to particular agents.

**Figure 10:**
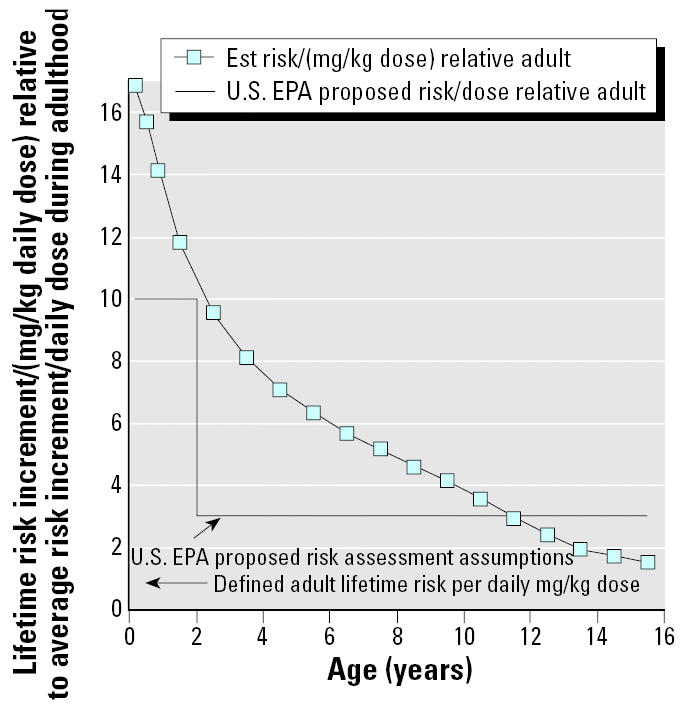
Summary of mean model predictions for the lifetime risk increment/(mg/kg dose-day) from constant mg/kg-day exposures of children of various ages (squares) compared with the U.S. EPA’s proposed assumptions (U.S. EPA 2003). Est, estimated.

The milligram per kilogram body weight^3/4^ scenario quantified in Table 9 represents a reasonable generic case for exposure via an environmental medium (e.g., air) whose intake depends on metabolism rates, which scale approximately with the three-quarters power of body weight. However, because many current risk assessments are done based on dosages expressed in milligrams per kilogram, rather than milligrams per kilogram body weight^3/4^, Table 10 and Figure 10 show comparable results for a scenario in which there is constant lifetime exposure in terms of simple milligrams per kilogram body weight^1^. This scenario also allows a direct comparison with expectations under the U.S. EPA proposal (EPA 2003) of factors of 10 and 3 for relative susceptibility per milligrams per kilogram dose for the first 2 years, and ages 2–15, respectively. Overall, Monte Carlo simulations using the constant milligrams per kilogram dosing produce a mean expected value for the lifetime risk that is 2.8 times what would be expected for adult-only exposure (compared with the 1.6 expected under the U.S. EPA proposal; U.S. EPA 2003) with 5–95% confidence limits of about 1.5–6 times the adult-only exposure risk. For the U.S. EPA’s 0- to 2-year and 2- to 15-year age groups (EPA 2003), we find mean expected risk increments of 13.7- and 4.7-fold relative to mean adult exposure risks, respectively. These are in the range of 35–55% larger than expected using the U.S. EPA’s proposed 10- and 3-fold factors (EPA 2003). Overall, these are not large differences, considering the relatively informal nature of the analysis underlying the U.S. EPA proposal; however, these results suggest that further studies may well suggest somewhat larger adjustments.

Both Tables 9 and 10 give results for each sex separately for completeness. However, to the degree that the sex-dependent differences in age-related susceptibility depend on sexual dimorphisms in CYP enzyme expression, readers should be cautioned that some CYP enzymes known to be expressed in a sex-related fashion in rats do not appear to correspond to CYP enzymes that are known to be present in humans (Mugford and Kedderis 1998). In the absence of direct human evidence that there are sex-related differences in age-specific susceptibility as substantial as those indicated in Tables 9 and 10, we recommend that risk assessors give most emphasis to the total population projections (both sexes combined) in evaluating the potential significance of early-life exposures.

There is one other “bottom line” inference that should be made clear. The results in Tables 9 and 10 directly imply that it is more likely than not that most of the total lifetime risk of cancers from continuous milligrams per kilogram-day or milligrams per kilogram^3/4^-day exposures to mutagenic carcinogens arises from exposures that are received before adulthood.

## Brief Discussion of Further Needs for Risk and Uncertainty Modeling to Estimate Full Life Mutagenic Cancer Risks for a Generic Example Chemical

Full application of these results to a real example chemical would ideally involve several additional steps: *a*) quantification of any differential exposure of children of various life stages relative to adults; *b*) integration of information from all available animal bioassays deemed acceptable for human risk projections; *c*) adjustments, if needed, for the inclusion of a portion of the “weaning to 60-day” period in the bioassays for the chemical, if the bioassays began exposures before our assumed 60-day starting point; *d*) integration of likelihood-based uncertainties in estimated dose–response slopes from the bioassay data into the overall uncertainty analysis; and *e*) incorporation of estimates of delivered dose, across species and life stages, preferably with the aid of physiologically based pharmacokinetic models, along with pharmacodynamic uncertainties in inter-species projections.

For example, data for induction of breast cancers by dimethylbenz[*a*]anthracene clearly indicate greater sensitivity in adolescent animals than either earlier or later in life (56% tumor incidence in 6–8 week animals, compared with 8% for females < 2 weeks of age and 15% for 26-week animals) (Ginsberg 2003; Meranze et al. 1969). [These specific data were not separately broken out by the U.S. EPA (2003) in its primary listing of data, and thus were included only in the form of a “total tumors” category in our original analysis (Hattis et al. 2004).] The observed age-dependent pattern of tumor induction in the breast is probably related to the cell division pattern in terminal end buds in the development of that tissue (Russo and Russo 1999). Human data for this parameter (Russo et al. 1987) might allow a greatly improved rodent-to-human equivalent age mapping for this tumor type.

## Conclusions

Improved life-stage–specific analyses are possible based on current information. These involve appreciable uncertainties, particularly in the mapping of rodent exposure periods to human equivalents. However, current understanding can at least provide decision makers and the public with preliminary estimates of the potential importance of exposures at early life stages in the overall context of cancer risks from genetically active agents. The suggestion of the present analysis is that early-life exposure could make important contributions to full-life cancer risks.

However, we offer the caveat that, because of the multistage and multifactor nature of cancer development, these analyses should be grounded on the mode of action of the specific agent or classes of agents with putatively similar modes of action. Specific agents affecting tumors at particular sites may also have different age patterns of sensitivity than the general run of mutagenic carcinogens represented in the present analysis. The present results are based on early-life sensitivity data for only nine chemicals, of which only five were classified as mutagenic. The conclusions about early-life sensitivity for carcinogens with different sites or modes of action could be altered as data become available for bioassays testing age-related differences in tumor risks after exposures to a broader set of chemicals.

## Figures and Tables

**Table 1:** Comparative results for male versus female animals for mutagenic chemicals: analysis of combined data from continuous and discrete dosing experiments (nine compounds, 153 tumor incidence observations).

Period	Maximum likelihood estimate of cancer inductions per dose/(body weight^3/4^-day)relative to comparably dosed adults	5–95% confidence limits	Arithmetic mean
Male animals			
Fetal	25	15.6–42	27
Birth to weaning	57	38–90	59
Weaning to 60 days	5.0	3.1–8.6	5.3
Female animals			
Fetal	1.77	1.05–2.9	1.83
Birth to weaning	4.4	3.3–6.0	4.5
Weaning to 60 days	0.82	0.50–1.29	0.85

Data from Hattis et al. (2004).

**Table 2:** Log(geometric mean) departures of age-related changes in susceptibility to carcinogenesis for individual mutagenic carcinogens from model “predictions”—combining all available cancer sites for each agent.*^a^*

	Log (observed/model predicted) cancer transformations/animal relative to adults		
Chemical	Fetal	Birth to weaning	Weaning to 60 days
Male animals			
Benzidine	0.004	0.163	–0.367
Benzo[*a*]pyrene	ND	0.017	–0.045
Deithylnitrosamine	–0.961	–0.045	0.132
DMBA	ND	–0.108	–0.166
Ethylnitrosourea	0.103	–0.135	0.423
*N*-Nitroso-*N*-methylurea	ND	–0.257	0.142
Safrole	–0.486	0.084	–0.236
Urethane	ND	0.008	–0.023
SD	0.490	0.132	0.249
Female animals			
Benzidine	–0.104	–0.111	ND
Benzo[*a*]pyrene	ND	0.037	0.098
Diethylnitrosamine	–1.086	–0.025	–0.172
DMBA	ND	–0.087	–0.038
Ethylnitrosourea	0.416	0.006	–0.142
*N*-Nitroso-*N*-methylurea	ND	–0.184	0.038
Safrole	ND	–0.720	ND
SD	0.763	0.269	0.115

Abbreviations: DMBA, dimethylbenz[*a*]anthracene; ND, no data.

a Data for only eight chemicals are shown, rather than the nine listed in Table 1, because for one chemical (vinyl chloride) there were no experimental groups where dosing was confined entirely to one of the three pre-adult periods represented here. Data combining exposures across periods and adulthood could contribute to the analysis for Table 1 because of the use of the dummy-variable analysis methodology described in Hattis et al. (2004).

**Table 3:** Age groupings recommended by the U.S. EPA for early-life exposure analyses.

Age groups < 1 year*^a^*	Age groups ≥1 year
Birth to < 1 month	1 to < 2 years
1 to < 3 months	2 to < 3 years
3 to < 6 months	3 to < 6 years
6 to < 12 months	6 to < 11 years
	11 to < 16 years
	16 to < 18 years
	18 to < 21 years to be considered on a case-by-case basis

a For evaluating exposure or potential dose but not internal dose, it may be acceptable to combine some of these groups (e.g., the first three groups could be combined to encompass “birth to < 6 months”). Data from Brennan et al. (2003).

**Table 4:** Species differences in times of beginning sexual maturity.

Species (time unit)	Male	Female
Mouse (months)	1.5	1.0
Rat (months)	1.8–2.1	1.8–2.1
Human (years)	11.5	10.5

Data from Kilborn et al. (2002).

**Table 5:** Mice: inferences of corresponding human ages from weight-based comparisons relative to the times of sexual maturity.

Time/event	Fraction of mouse weight at sexual maturity	Source of human weight data	Corresponding human age	Unit of human age
Male mouse				
Begin fetal dosing (GD12)	6.4 × 10^–4^	Potter and Craig 1975	93	Gestation days
Birth (GD20)	0.048	Potter and Craig 1975	35	Gestation weeks
Weaning (PND21)	0.354	NHANES III	3.16	Postnatal years
Adult (PND60)	1.163	NHANES III	12.8	Postnatal years
Female mouse				
Begin fetal dosing (GD12)	1.8 × 10^–3^	Potter and Craig 1975	112	Gestation days
Birth (GD20)	0.092	Sunderman and Boerner 1949	14	Postnatal days
Weaning (PND21)	0.677	NHANES III	7.40	Postnatal years
Adult (PND60)	1.435	NHANES III	15.1	Postnatal years

Abbreviations: GD, gestation day; PND, postnatal day.

**Table 6:** Rats: inferences of corresponding human ages from weight-based comparisons relative to the times of sexual maturity.

Time/event	Fraction of mouse weight at sexual maturity	Source of human weight data	Corresponding human age	Unit of human age
Male rat				
Begin fetal dosing (GD12)	6.3 × 10^–5^	Potter and Craig 1975	66	Gestation days
Birth (GD22)	0.023	Potter and Craig 1975	28	Gestation weeks
Weaning (PND21)	0.195	NHANES III	0.44	Postnatal years
Adult (PND60)	1.035	NHANES III	11.7	Postnatal years
Female rat				
Begin fetal dosing (GD12)	6.3 × 10^–5^	Potter and Craig 1975	66	Gestation days
Birth (GD22)	0.029	Potter and Craig 1975	30	Postnatal weeks
Weaning (PND21)	0.250	NHANES III	0.90	Postnatal years
Adult (PND60)	1.025	NHANES III	10.6	Postnatal years

Abbreviations: GD, gestation day; PND, postnatal day.

**Table 7:** Estimated lengths of various life stages in humans inferred from the ages of sexual maturity in mice, rats, and humans, and patterns of growth of body weight for rodents through 60 days of age, and for humans through 16 years of age.

Rodent sex and life-stage equivalent	Mouse-based estimate (days)	Rat-based estimate (days)	Geometric mean (days)	Geometric SD
Males				
GD12 to birth (fetal)	150	134	142	1.11
Birth to weaning	1,180	235	527	3.94
Weaning to 60 days	3,510	4,130	3,810	1.15
Females				
GD12 to birth (fetal)	175	142	157	1.20
Birth to weaning	2,690	392	1,030	5.12
Weaning to 60 days	2,830	3,560	3,170	1.22

GD, gestation day. All data for this table were rounded to three significant figures. This overstates the likely accuracy of the underlying projections. However, three significant figures are retained here to allow reasonably accurate reproduction of our later calculations by other analysts.

**Table 8:** Detailed results by life stage and sex: uncertainty distributions of risks for full lifetime exposures to a generic mutagenic carcinogen at a constant dose rate per kilogram body weight^3/4^.

	Risk relative to adult period		
Percentile of uncertainty distribution	Fetal	Birth to weaning	Weaning to 60-day
Males			
1	0.011	0.054	0.167
2.5	0.018	0.084	0.217
5	0.026	0.135	0.273
10	0.039	0.220	0.365
25	0.078	0.565	0.563
50	0.173	1.44	0.882
75	0.392	3.77	1.38
90	0.764	7.79	2.03
95	1.20	10.7	2.53
97.5	1.72	13.2	3.18
99	2.89	17.4	3.91
Arithmetic mean	0.351	2.92	1.09
Females			
1	0.000	0.004	0.012
2.5	0.000	0.007	0.021
5	0.001	0.012	0.034
10	0.001	0.023	0.049
25	0.004	0.069	0.074
50	0.014	0.210	0.107
75	0.047	0.564	0.150
90	0.137	1.09	0.199
95	0.278	1.57	0.233
97.5	0.505	2.10	0.273
99	0.961	2.79	0.323
Arithmetic mean	0.072	0.432	0.118

The numbers represent the increment to lifetime relative risk/dose where the risk from treatment for the full adult period is defined as 1.

**Table 9:** Overall results for constant mg/kg body weight^3/4^ dosing: uncertainty distributions of full lifetime risks for lifetime exposures to a generic mutagenic carcinogen at a constant dose rate per kilogram body weight^3/4^.

	Full lifetime risk relative to adult period only		
Percentile of uncertainty distribution	Male	Female	Male and female population
1	1.71	1.19	1.45
2.5	1.87	1.20	1.53
5	2.04	1.22	1.63
10	2.28	1.25	1.76
25	2.91	1.31	2.11
50	4.10	1.46	2.78
75	6.51	1.78	4.15
90	10.2	2.33	6.26
95	13.0	2.77	7.89
97.5	15.9	3.33	9.62
99	19.5	4.06	11.8
Arithmetic mean	5.38	1.66	3.52

**Table 10:** Overall results for constant mg/kg body weight^1^ exposures: uncertainty distributions of full lifetime incremental risks for lifetime exposures to a generic mutagenic carcinogen at a constant dose rate per kilogram body weight^1^.

	Full lifetime risk relative to adult period only		
Percentile of uncertainty distribution	Male	Female	Male and female population
1	1.53	1.15	1.34
2.5	1.65	1.16	1.41
5	1.76	1.18	1.47
10	1.92	1.19	1.56
25	2.34	1.24	1.79
50	3.19	1.34	2.27
75	4.85	1.58	3.22
90	7.54	1.98	4.76
95	9.69	2.34	6.02
97.5	11.7	2.72	7.19
99	14.5	3.43	8.98
Arithmetic mean	4.10	1.50	2.80
